# Role of enzymatic activity in muscle damage and cytotoxicity induced by *Bothrops asper* Asp49 phospholipase A_2_ myotoxins: are there additional effector mechanisms involved?

**DOI:** 10.7717/peerj.569

**Published:** 2014-09-16

**Authors:** Diana Mora-Obando, Cecilia Díaz, Yamileth Angulo, José María Gutiérrez, Bruno Lomonte

**Affiliations:** 1 Instituto Clodomiro Picado, Facultad de Microbiologia, Universidad de Costa Rica, San José, Costa Rica; 2 Departamento de Bioquímica, Escuela de Medicina, Universidad de Costa Rica, San José, Costa Rica

**Keywords:** Snake venom, Asp49, Myotoxin, Phospholipase A_2_, *Bothrops asper*

## Abstract

Viperid venoms often contain mixtures of Asp49 and Lys49 PLA_2_ myotoxin isoforms, relevant to development of myonecrosis. Given their difference in catalytic activity, mechanistic studies on each type require highly purified samples. Studies on Asp49 PLA_2_s have shown that enzyme inactivation using *p*-bromophenacyl bromide (*p*-BPB) drastically affects toxicity. However, based on the variable levels of residual toxicity observed in some studies, it has been suggested that effector mechanisms independent of catalysis may additionally be involved in the toxicity of these enzymes, possibly resembling those of the enzymatically inactive Lys49 myotoxins. A possibility that Lys49 isoforms could be present in Asp49 PLA_2_ preparations exists and, if undetected in previous studies, could explain the variable residual toxicity. This question is here addressed by using an enzyme preparation ascertained to be free of Lys49 myotoxins. In agreement with previous reports, inactivation of the catalytic activity of an Asp49 myotoxin preparation led to major inhibition of toxic effects *in vitro* and *in vivo*. The very low residual levels of myotoxicity (7%) and cytotoxicity (4%) observed can be attributed to the low, although detectable, enzyme remaining active after *p*-BPB treatment (2.7%), and would be difficult to reconcile with the proposed existence of additional catalytic-independent toxic mechanisms. These findings favor the concept that the effector mechanism of toxicity of Asp49 PLA_2_ myotoxins from viperids fundamentally relies on their ability to hydrolyze phospholipids, arguing against the proposal that membrane disruption may also be caused by additional mechanisms that are independent of catalysis.

## Introduction

Phospholipases A_2_ (PLA_2_s) are abundant components of snake venoms, where they play major toxic roles in the immobilization and/or killing of prey. Among their diverse effects, these interfacial enzymes exert potent neurotoxicity and/or myotoxicity, and efforts to reveal their detailed mechanisms of action on neurons and skeletal muscle cells have long been pursued in toxinology (Kini, 1997; Gutiérrez & Lomonte, 2013). PLA_2_ enzymes found in Elapidae and Viperidae snake venoms conserve a common scaffold but present distinctive structural characteristics which allow their classification within groups I or II, respectively, of this large protein family (Arni & Ward, 1996; Montecucco, Gutiérrez & Lomonte, 2008). Since two different, non-toxic ancestral PLA_2_ genes evolved toward toxicity in elapids (group I) and viperids (group II) (Fry & Wüster, 2004), the acquisition of common toxic activities in both lineages represents a convergent evolutionary process (Lomonte & Gutiérrez, 2011; Lomonte & Rangel, 2012). As a consequence, mechanistic differences might exist in the mode by which group I and group II PLA_2_s exert their shared toxic effects. Furthermore, within the group II enzymes a subtype emerged which is typified by the substitution of the critical Asp49 residue in the catalytic center, most often by a Lys residue (Maraganore et al., 1984). Such Lys49 PLA_2_s are indeed “PLA_2_ homologues” or “PLA_2_-like” proteins because they are devoid of phospholipolytic activity, but are highly active as myotoxins. All Lys49 PLA_2_ homologues studied to date are basic proteins that induce skeletal muscle necrosis at the site of injection (Lomonte, Angulo & Calderón, 2003; Lomonte et al., 2009; Lomonte & Rangel, 2012), whereas not all Asp49 PLA_2_s from viperids are myotoxic. This effect is present almost exclusively in the basic but not the acidic enzymes, in spite of their shared catalytic activity (Lomonte & Gutiérrez, 2011).

Myotoxic PLA_2_s and PLA_2_ homologues present in viperid venoms have increasingly been studied due to their relevance in the development of skeletal muscle necrosis after envenomings, with serious clinical consequences such as tissue loss, dysfunction or amputation (Harris & Cullen, 1990; Gutiérrez & Ownby, 2003; Soares, Fontes & Giglio, 2004; Lomonte & Rangel, 2012). In order to understand the mechanisms involved in the pathological effects induced by group II myotoxic PLA_2_s, thorough protein purification is essential. The isolation of different variants of myotoxic PLA_2_s in viperid venoms is complicated by the fact they often contain a multiplicity of closely related isoforms that are variably expressed in the species (Faure & Bon, 1988; Lomonte & Kahan, 1988; Valiente et al., 1992) or in single individuals (Lomonte & Carmona, 1992). As a consequence, these venoms may contain complex mixtures of both Asp49 and Lys49 myotoxins, and given the fundamental difference in catalytic activity between them, mechanistic studies on each type require their efficient separation. Contamination of a Lys49 myotoxin preparation with Asp49 PLA_2_s can be straightforwardly detected by means of the catalytic activity of the latter (Scott et al., 1992; Cintra-Francischinelli et al., 2009). However, the converse situation poses a more difficult challenge, since there is no specific functional assay to detect the presence of Lys49 contaminants in preparations of Asp49 myotoxins.

Several Asp49 and Lys49 myotoxins have been isolated from the venom of *Bothrops asper*, the snake species causing the majority of envenomings in Central America (Angulo & Lomonte, 2009). A number of studies have demonstrated that Lys49 PLA_2_ homologues induce myonecrosis by a catalytic-independent mechanism, involving amino acids at their C-terminal region which affect the integrity of the sarcolemma (Lomonte et al., 1994; Núñez, Angulo & Lomonte, 2001; Chioato et al., 2002; Chioato & Ward, 2003; Lomonte, Angulo & Calderón, 2003; dos Santos et al., 2009; Lomonte & Rangel, 2012; Fernandes et al., 2013; Fernández et al., 2013). On the other hand, Asp49 PLA_2_s have been shown to depend on their catalytic activity to disrupt the integrity of this membrane (Lomonte & Gutiérrez, 2011). Nevertheless, some studies have reported that inhibition of PLA_2_ activity in Asp49 myotoxins may result in variable degrees of residual toxicity, and the possibility has been suggested that their overall toxic action may involve membrane-perturbing mechanisms additional to catalysis depending on molecular regions other than the catalytic site, in similarity with the Lys49 myotoxins (Díaz et al., 1991; Díaz-Oreiro & Gutiérrez, 1997; Andrião-Escarso et al., 2000; Soares et al., 2001a; Soares et al., 2001b; Soares & Giglio, 2003). An alternative explanation for the residual toxicity observed in some earlier studies after enzyme inactivation of Asp49 PLA_2_s, could be the presence of variable levels of undetected Lys49 isoforms. The present work addresses this possibility by reassessing the role of catalytic activity in the toxic effects of an Asp49 PLA_2_ myotoxin preparation from the venom of *B. asper* that was ascertained free of Lys49 myotoxin contaminants. After its inactivation by histidine modification with *p*-bromophenacyl bromide, its toxic actions *in vivo* (myotoxicity) and *in vitro* (cytotoxicity upon differentiated myotubes) were evaluated in comparison to the untreated toxin.

## Methods

### Isolation of Asp49 phospholipase A_2_ myotoxins

Venom of *Bothrops asper*, pooled from specimens collected in the Pacific versant of Costa Rica and kept at the serpentarium of Instituto Clodomiro Picado, was fractionated by cation-exchange chromatography on a CM-Sephadex C-25 column (20 × 2 cm) equilibrated with 0.05 M Tris, 0.1 M KCl, pH 7.0 buffer, at 0.4 mL/min (Lomonte & Gutiérrez, 1989). Protein elution was performed with a gradient toward 0.75 M KCl in the same buffer, and monitored at 280 nm with a Bio-Logic chromatograph (Bio-Rad). Peak 2 was collected, desalted by overnight dialysis, lyophilized, and further separated by reverse-phase high-performance liquid chromatography (RP-HPLC) on a C_8_ semipreparative column (250 × 10 mm) Elution was carried out at 2.5 mL/min with a gradient from water to acetonitrile, both containing 0.1% trifluoroacetic acid, in a model 1200 chromatograph (Agilent) monitored at 215 nm. The gradient toward acetonitrile stepped from 0 to 25% in 5 min, 25 to 50% in 22 min, 50 to 70% in 1 min, and was sustained at 70% for 2 min, for a total run time of 30 min. Fractions of interest were collected, dried in a vacuum centrifuge at 45 °C, and stored at −20 °C. Protein concentrations were estimated by measuring their absorbance at 280 nm in a Nanodrop (Thermo) reader.

### Homogeneity evaluation

The Asp49 PLA_2_ preparation was evaluated by three analytical methods. **(a)** A sample (40 µg) of the fraction was run by RP-HPLC on a C_4_ column at analytical scale (4.6 × 150 mm), eluted at 1 mL/min with an acetonitrile gradient (0% at 5 min, 0 to 70% in 45 min, and 70% for 10 min, for a total run time of 60 min) and monitored at 215 nm; **(b)** the fraction was analyzed by nano-electrospray mass spectrometry (nESI-MS), by directly infusing the sample (2 µg/10 µL), loaded into a metal-coated capillary tip (Proxeon), in a Q-Trap 3200 instrument (Applied Biosystems) operated in positive multicharge enhanced mode using an ionization voltage of 1,200–1,300 V. Spectra were acquired in the 700–1,700 m/z range and deconvoluted with the BioAnalyst v.1.5 software (ABSciex); **(c)** the fraction (200 pmoles) was subjected to ten cycles of N-terminal sequencing by automated Edman degradation in a model PPSQ33A instrument (Shimadzu Biotech). In all three analytical techniques, a sample of Lys49 myotoxin (*B. asper* myotoxin II; Lomonte & Gutiérrez, 1989), was included for comparison.

### Asp49 phospholipase A_**2**_ inactivation with *p*-bromophenacyl bromide

Three mg of the Asp49 myotoxin were dissolved in 1 mL of 0.1 M Tris, 0.7 mM EDTA, pH 8.0 buffer. Then, 125 µL of *p*-bromophenacyl bromide (*p*-BPB; 1.5 mg/mL in ethanol; Sigma) were added and incubated at room temperature (20–25 °C) for 24 h (Díaz-Oreiro & Gutiérrez, 1997). Excess reagent and salts were eliminated by RP-HPLC on a semi-preparative C_8_ column, as described above. The protein was collected and finally dried by vacuum centrifugation at 45 °C. To serve as a control in all assays, a parallel sample of the Asp49 PLA_2_ was subjected to all steps of this procedure, but omitting the *p*-BPB reagent during the 24 h incubation.

### Phospholipase A_**2**_ activity

PLA_2_ activity was assayed on the synthetic monodisperse substrate 4-nitro-3-octanoyloxybenzoic acid (NOBA; Holzer & Mackessy, 1996). Various amounts of *p*-BPB-treated or control myotoxin, dissolved in 25 µL of 10 mM Tris, 0.1 M NaCl, 10 mM CaCl_2_, pH 8.0 buffer, were mixed with 200 µL of this buffer and 25 µL of NOBA (1 mg/mL in acetonitrile), and incubated at 37 °C for 60 min (Mora-Obando et al., 2014). The reaction product was colorimetrically quantified at 405 nm in a microplate reader (Thermo). Wells containing all reagents, except the enzyme, were used as a blank. All samples were assayed in triplicate wells.

### Cytotoxic activity

Cytotoxic activity was assayed on the murine myogenic cell line C2C12 (ATCC-CRL1772) (Lomonte et al., 1999). Various amounts of *p*-BPB-treated or control myotoxin, dissolved in 150 µL of assay medium (Dulbecco’s modified Eagle’s medium with 1% fetal calf serum [DMEM, 1% FCS]), were added to wells of a 96-well plate where the myogenic cells had grown until confluency and then differentiated to myotubes for 4–6 days in assay medium at 37 °C and 7% CO_2_. The medium was removed and, immediately, the toxins dissolved in a fresh assay medium were added. After 3 h of incubation at 37 °C, an aliquot of cell supernatants (60 µL) was collected from each well and the released lactate dehydrogenase (LDH) activity was quantified by a UV kinetic assay (LDH-BR Cromatest; Linear Chemicals). Controls for 0 and 100% cytotoxicity consisted of assay medium and 0.1% Triton X-100 diluted in assay medium, respectively. All samples were assayed in triplicate wells.

### Myotoxic activity

Myotoxic activity was assayed in groups of five CD-1 mice of 18–20 g of body weight. These *in vivo* assays followed protocols approved by the Institutional Committee for the Use and Care of Animals (CICUA; 132-13), University of Costa Rica. Mice were housed in cages in groups of 4–6, and provided food and water *ad libitum*. A fixed amount of the toxins (50 µg), dissolved in 50 µL of phosphate-buffered saline (PBS; 0.12 M NaCl, 0.04 M sodium phosphate buffer, pH 7.2), was injected intramuscularly into the gastrocnemius (Lomonte & Gutiérrez, 1989). A control group of mice received an identical injection of PBS only. After 3 h, blood was collected from the tip of the tail into a heparinized capillary and centrifuged. The plasma creatine kinase (CK) activity, expressed in U/L, was determined using a UV kinetic assay (CK-Nac; Biocon Diagnostik).

### Statistical analysis

Statistical significance of mean values comparisons was determined by the Student’s *t*-test, at *p* < 0.05, using the GraphPad Instat v.3 software.

## Results and Discussion

**Figure 1:**
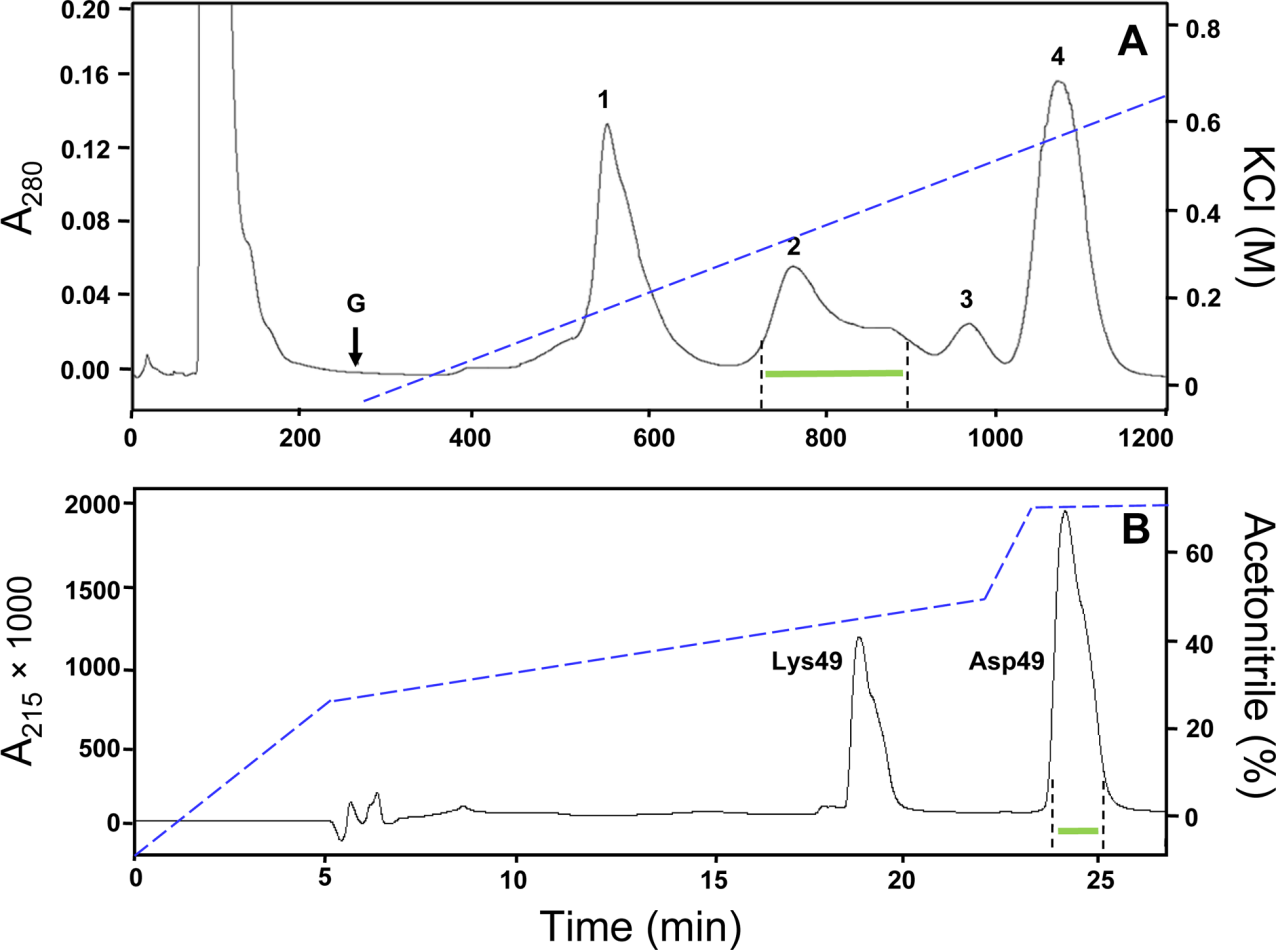
Isolation of myotoxic phospholipases A_2_ from the venom of *Bothrops asper* (Pacific versant of Costa Rica). (A) Venom (200 mg) was applied to a CM-Sephadex C-25 column (20 × 2 cm) equilibrated with 0.05 M Tris, 0.1 M KCl, pH 7.0 and, after elution of unbound proteins, a linear gradient (G; dotted line) toward 0.05 M Tris, 0.75 M KCl, pH 7.0 buffer was developed. Proteins from peak 2 (horizontal green line) were pooled and further separated by (B) reverse-phase HPLC on a C_8_ semipreparative column (250 × 10 mm). Proteins were eluted at 2.5 mL/min with a gradient from water to acetonitrile (dotted line) in the presence of 0.1% trifluoroacetic acid. The second major peak (∼24 min), corresponding to Asp49 phospholipases A_2_, was collected and subjected to further analyses.

Fractionation of *B. asper* (Pacific region of Costa Rica) venom on CM-Sephadex at pH 7.0 separated the basic proteins from the acidic components eluting in the flow-through peak (Fig. 1A). Peak 1 corresponded to the metalloproteinase BaP1 described by Gutiérrez et al. (1995), whereas peaks 2–4 contained basic PLA_2_s or PLA_2_ homologues, in similarity with the profile of *B. asper* venom from specimens of the Caribbean region (Gutiérrez, Ownby & Odell, 1984; Lomonte & Gutiérrez, 1989). Although the resolution obtained in the Asp49-rich region of the Pacific venom chromatogram was slightly lower in comparison to the Caribbean venom, the final yield of Asp49 PLA_2_ was found to be higher with the former venom, which was therefore selected for further purification. Peak 2 (Fig. 1A) presented high PLA_2_ activity (data not shown) and was subsequently fractionated by semi-preparative C_8_ RP-HPLC, resulting in two major peaks (Fig. 1B). The peak eluting at ∼19 min was devoid of PLA_2_ activity, corresponding to Lys49 myotoxins, whereas the larger peak eluting at ∼24 min showed PLA_2_ activity, corresponding to Asp49 myotoxins.

**Figure 2:**
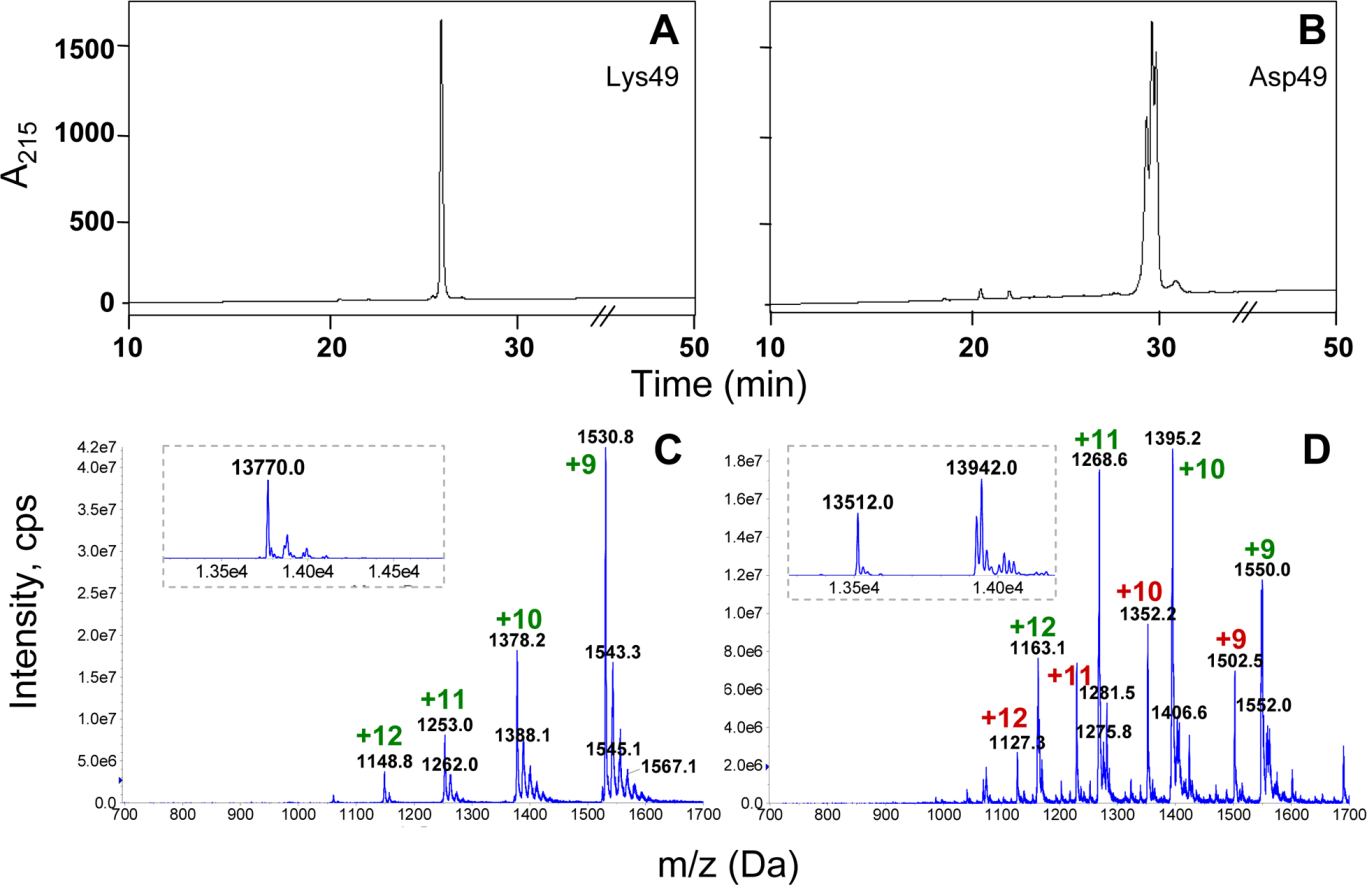
Analysis of the Asp49 phospholipase A_2_ preparation obtained after cation-exchange chromatography and semi-preparative RP-HPLC on C_8_. A Lys49 myotoxin control (A) and the Asp49 peak shown in Fig. 1 (B) were subjected to analytical RP-HPLC on a C_4_ column, using a 60 min gradient (see Materials and Methods). For clarity, the gradient line is omitted and the time scale is magnified in the region of interest. The same samples were analyzed by nano-electrospray mass spectrometry in (C) and (D). Insets within dotted lines show the corresponding deconvolutions of the multicharged ion series and calculation of the isotope-averaged observed molecular masses of these samples.

In order to ascertain that the Asp49 PLA_2_ preparation was devoid of Lys49 proteins, several analyses were conducted. First, the sample was subjected to analytical RP-HPLC using a C_4_ column with a longer elution gradient. In similarity with the semi-preparative C_8_ separation, this technique efficiently separated the Lys49 from the Asp49 proteins with a difference in retention times between them of nearly 3 min (Figs. 2A and 2B). The higher resolution of this technique evidenced that the Asp49 PLA_2_ preparation contained several peaks with very close retention times, which could not be adequately resolved (Fig. 2B). In contrast, the Lys49 protein eluted as a sharp single peak (Fig. 2A). nESI-MS analyses confirmed these observations, revealing the presence of at least two main molecular masses of 13,512 ± 2 and 13,942 ± 2 Da in the Asp49 preparation (Fig. 2D), while the Lys49 myotoxin presented a main mass of 13,770 ± 3 Da (Fig. 2C). The latter mass is in agreement with that expected for myotoxin II from the Caribbean versant of Costa Rica (UniProt accession; P24605; M_*av*_ =13773), if the Leu/Phe ambiguity reported for its position 114 (Francis et al., 1991) is considered as Phe (the sequence P24605 in databanks shows Leu at this position).

**Figure 3:**
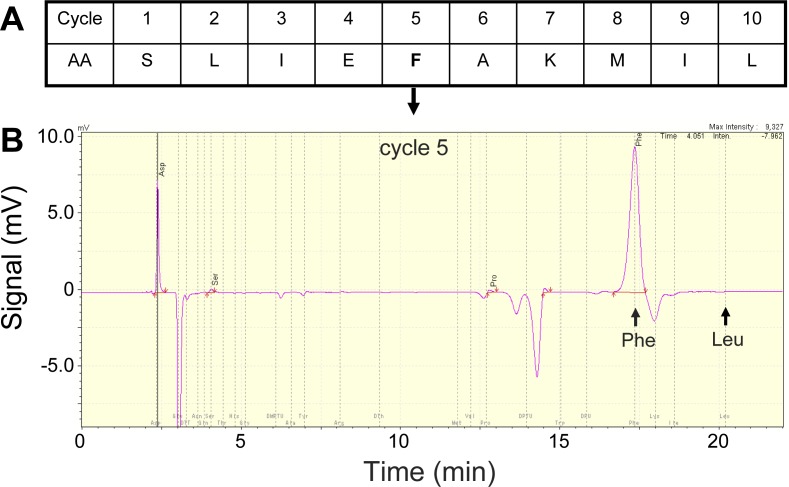
N-terminal amino acid sequencing of the Asp49 phospholipase A2 N-terminal amino acid sequencing of the Asp49 phospholipase A2 peak shown in Fig. 1 (A; first ten residues), and snapshot of the fifth cycle chromatogram (B). As expected for this type of enzyme, this cycle yields a strong signal corresponding to phenylalanine (Phe), while the retention time corresponding to leucine (Leu), a conserved position in Lys49 myotoxins, shows no traces of this amino acid residue.

Since both the semi-preparative and analytical RP-HPLC procedures were able to resolve Lys49 from Asp49 proteins of this venom, it was assumed that the heterogeneity observed in the latter preparation would correspond only to Asp49 isoforms (free of Lys49 contamination). In order to evaluate this assumption, the Asp49 PLA_2_ sample was subjected to ten cycles of automated N-terminal sequencing. The resulting sequence showed single amino acid signals in all cycles but, most importantly, showed no traces of Leu in the fifth cycle (Fig. 3). Since this amino acid position is conserved in all known Lys49 myotoxins, this result confirmed that the RP-HPLC yielded an Asp49 PLA_2_ preparation that, despite containing isoforms possibly due to microheterogeneity, should be free from any contaminating Lys49 PLA_2_ homologues. The short N-terminal sequence obtained (Fig. 3) matches with 10/10 identity the Asp49 PLA_2_ myotoxin reported by Kaiser et al. (1990), which has been variably described in the literature as *B. asper* myotoxin I or myotoxin III (UniProt accession P20474). This is the only basic Asp49 PLA_2_ myotoxin isolated from *B. asper* venom that has been sequenced, but it should be noted that it was reported as containing ambiguous amino acid residues in at least three positions near its C-terminus (Kaiser et al., 1990), evidencing the difficulties in resolving such closely related isoforms during isolation. Due to this complex microheterogeneity of isoforms, we made no attempts to assign the masses of the purified Asp49 protein preparation (13,512 ± 2 and 13,942 ± 2 Da) to any variable combination of the Asp49 myotoxin sequence previously reported (Kaiser et al., 1990), but instead refer to these proteins only as Asp49 PLA_2_ myotoxins.

The final Asp49 PLA_2_ preparation was subjected to histidine modification using *p*-BPB, aiming to reassess earlier studies on the role of enzyme activity in its toxic activities by ruling out the possibility of the presence of Lys49 contamination. The difference with previous studies (Díaz et al., 1991; Bultrón, Thelestam & Gutiérrez, 1993; Díaz-Oreiro & Gutiérrez, 1997) is that Asp49 PLA_2_ myotoxin isolation was earlier accomplished only by cation-exchange chromatography without the further use of HPLC and screening by sensitive analytical techniques such as mass spectrometry. Therefore the possible presence of undetected levels of Lys49 myotoxins remained open, in contrast to the present work.

**Figure 4:**
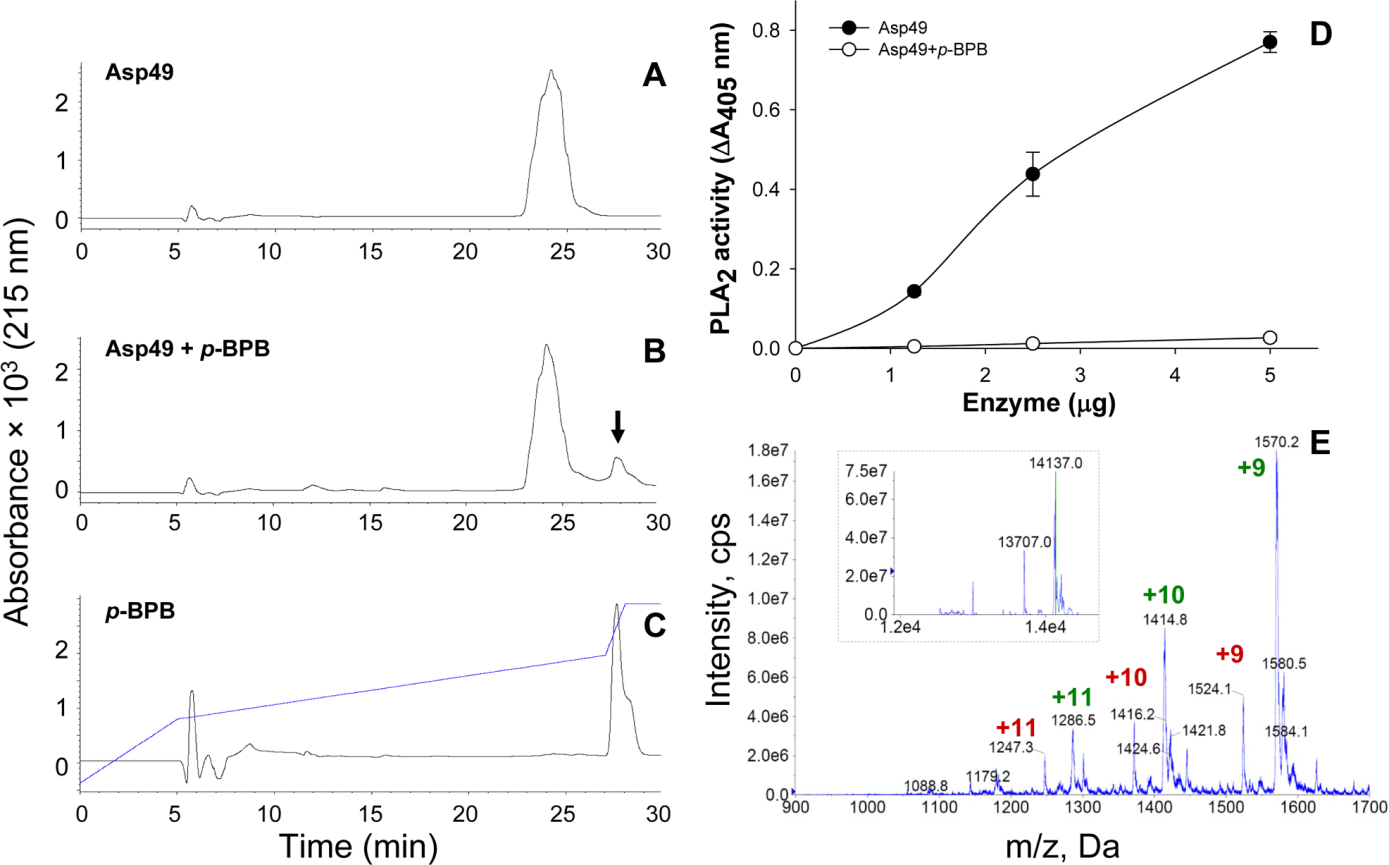
Modification of the Asp49 phospholipase A_2_ by *p*-bromophenacyl bromide (*p*-BPB). (A) unmodified enzyme control; (B) *p*-BPB-treated enzyme; and (C) *p*-BPB reagent control. The three samples were separated by RP-HPLC in a semi-preparative C_8_ column as in Fig. 1B. (D) Comparison of the phospholipase A_2_ (PLA_2_) activities of the control Asp49 enzyme and the *p*-BPB-treated enzyme on the synthetic monodisperse substrate 4-nitro-3-octanoyloxybenzoic acid. Each point represents mean ± SD of three replicates. (E) Nano-electrospray mass spectrometry confirmation of the covalent incorporation of a single molecule of *p*-BPB in the modified Asp49 myotoxin. The observed isotope-averaged masses of the modified Asp49 preparation show an increase of 195 ± 3 Da in comparison to the masses of untreated proteins (Fig. 2D).

As shown in Figs. 4A–4C, the *p*-BPB-modified Asp49 PLA_2_ did not alter its retention time in comparison to the control toxin, and was readily separated from the excess reagent by the C_8_ RP-HPLC procedure. The covalent incorporation of a single molecule of *p*-BPB was confirmed by nESI-MS (Fig. 4E), where an increase in molecular mass of 195 ± 3 Da (0.02% instrumental error) was observed in comparison to the unmodified protein (Fig. 2D), in agreement with the theoretically expected mass increase of 198 Da as a result of this reaction. This covalent modification should correspond to the single His48 residue of the protein (consensus numbering), which inactivates the catalytic mechanism of PLA_2_s (Volwerk, Pieterson & de Haas, 1974). Inactivation of catalysis was confirmed (Fig. 4D), although the use of a sensitive synthetic substrate allowed the detection of a low level of residual PLA_2_ activity at the highest protein concentration tested, corresponding to ∼2.7% of the unmodified enzyme. The difference between this result and previous studies where PLA_2_ activity was reported to be undetectable (0%) after 24 h of reaction with *p*-BPB, using similar protocols, may reside in the different sensitivities of the enzymatic assays used.

**Figure 5:**
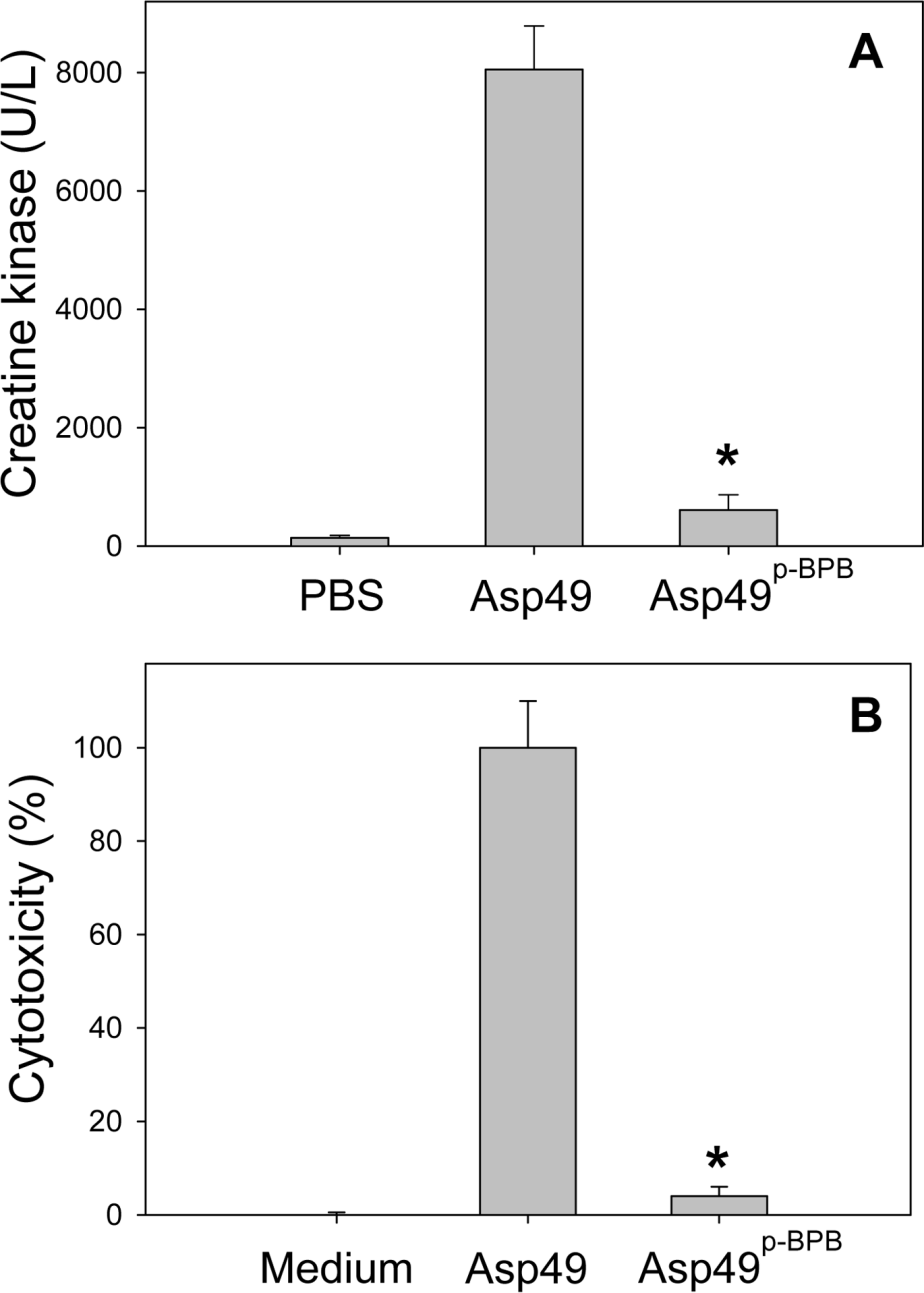
Comparison of the myotoxic (A) and cytotoxic (B) activities of untreated and *p*-BPB-treated Asp49 myotoxins. Bars in (A) represent mean ± SD of five mice. Values corresponding to *p*-BPB-modified enzyme are very low, although significantly higher (*; *p* < 0.05) in comparison to the phosphate-buffered saline (PBS) in (A) or culture medium in (B). Bars represent mean ± SD of five replicates in (A) or three replicates in (B).

The *p*-BPB-modified Asp49 PLA_2_ was tested for myotoxicity in mice (Fig. 5A) and for *in vitro* cytotoxicity upon differentiated C2C12 myotubes (Fig. 5B), in comparison to the unmodified toxin control. Results showed that inactivation of catalytic activity led to a major inhibition of both toxic effects, in agreement with the previous observations of Díaz-Oreiro & Gutiérrez (1997). The very low residual levels of toxicity observed can be attributed to the low, but detectable amounts of enzyme that remained catalytically active after *p*-BPB treatment. Some studies have reported variable levels of residual toxicity for Asp49 myotoxins under conditions that abrogated PLA_2_ activity by using *p*-BPB (Table 1). For example, residual toxicities of 30%, 14% and 16%, were observed in studies with *B. asper* myotoxin III, crotoxin B, and piratoxin III, respectively after *p*-BPB modification (Table 1). These observations have led to the suggestion that viperid Asp49 PLA_2_ myotoxins might possess mechanisms additional to their catalytic activity, to exert toxicity (Díaz et al., 1991; Bultrón, Thelestam & Gutiérrez, 1993; Díaz-Oreiro & Gutiérrez, 1997; Andrião-Escarso et al., 2000; Soares et al., 2001a; Soares et al., 2001b). The present results strengthen the concept that the effector mechanism of toxicity of viperid Asp49 PLA_2_ myotoxins, i.e., the capacity to disrupt the integrity of plasma membrane, essentially relies on their ability to hydrolyze phospholipids on the membrane of target cells (Fernández et al., 2013). On the other hand, *p*-BPB, besides blocking the catalytic center of Asp49 PLA_2_s, may induce subtle conformational changes at distant sites. Such changes have been shown by crystallography in the bovine pancreatic PLA_2_ (Renetseder et al., 1988) and the non-myotoxic, acidic PLA_2_ BthA-I from *Bothrops jararacussu* venom (Magro et al., 2005), although not in the case of the acidic APLA_2_ from *Agkistrodon halys* venom (Zhao et al., 1998). Moreover, conformational changes induced by *p*-BPB have also been demonstrated for two Lys49 myotoxins, PrTX-I from *B. pirajai* (Marchi-Salvador et al., 2009) and BthTX-I from *B. jararacussu* (Fernandes et al., 2010). These myotoxins, in spite of being enzymatically inactive, suffer a reduction in myotoxicity of nearly 50% when modified by *p*-BPB, which has been possibly attributed to the conformational changes caused by the alkylation of their absolutely conserved His48 (Díaz et al., 1993; Andrião-Escarso et al., 2000; Soares et al., 2000; Marchi-Salvador et al., 2009). In contrast, as shown by the present results and some previous studies (Table 1), the Asp49 myotoxins behave differently since their toxicity is essentially lost after His48 alkylation, concomitantly with the loss of catalytic activity. Since conformational changes induced by His48 alkylation do not completely abrogate toxicity in the case of Lys49 myotoxins, but leave a residual effect as high as 50%, the complete loss of toxicity in *p*-BPB-treated Asp49 myotoxins would be hard to reconcile with the proposal that mechanisms independent of catalysis (in a manner analogous to the catalytic-independent membrane-damaging action of Lys49 myotoxins) may also be responsible for muscle necrosis. In line with this concept, it has been shown that synthetic peptides from the C-terminal region of Asp49 PLA_2_ myotoxins do not display toxicity, in contrast to equivalent peptides from Lys49 myotoxins which reproduce the catalytic-independent toxic effect of their parent molecules (Núñez, Angulo & Lomonte, 2001; Lomonte, Angulo & Santamaría, 2003; Cintra-Francischinelli et al., 2010).

**Table 1:** Summary of studies. The inactivation of group II Asp49 phospholipases A_2_ (PLA_2_s) from snake venoms by *p*-bromophenacyl bromide (*p*-BPB) and its consequences for myotoxicity *in vivo* or cytotoxicity *in vitro* have been analyzed.

Asp49 PLA_2_	Residual PLA_2_ activity (%)	Residual toxicity (%), assay	Reference
*Crotalus d. terrificus* crotoxin-B	15	very low myotoxicity^b^	Gopalakrishnakone et al. (1984)
*Bothrops asper* myotoxin III	3	30, cytotoxicity	Bultrón, Thelestam & Gutiérrez (1993)
*Bothrops asper* myotoxin III	0	4, myotoxicity	Díaz-Oreiro & Gutiérrez (1997)
*Lachesis muta* LM-PLA_2_	0^a^	0, myotoxicity	Fuly et al. (2000)
*Bothrops jararacussu* BthTX-II	0.05	1, cytotoxicity	Andrião-Escarso et al. (2000)
		0.5, myotoxicity	
*Crotalus d. terrificus* crotoxin-B	0	14.1, myotoxicity	Soares et al. (2001a)
	5.9^a^		
*Bothrops jararacussu* BthTX-II	0	5, myotoxicity	Soares & Giglio (2003)
		1, cytotoxicity	
*Bothrops pirajai* PrTX-III	0	16, myotoxicity	Soares et al. (2001b)
		5, cytotoxicity	
*Bothrops asper* Asp49 myotoxins	2.7	7, myotoxicity	Present study
		4, cytotoxicity	

**Notes.**

a Determined by indirect hemolysis.

b Analyzed qualitatively, and described as “minimal myotoxicity”.

On the other hand, in the case of elapid PLA_2_s (group I), a recent study on notexin and site-directed mutants has shown that the catalytically-inactive Asp49/Lys mutant retains a cytotoxic activity as high as 35–50% of that corresponding to the wild-type protein (Simonato et al., 2014), indicating that in addition to its catalytic-dependent toxic mechanism, it also exerts toxicity by a yet uncharacterized catalytic-independent action. Thus, elapid PLA_2_s may present important differences with viperid PLA_2_s regarding structure–function relationships and toxic mechanisms.

Altogether, current evidence on the mode of action of the two divergent subtypes of group II PLA_2_ myotoxins of viperids supports the notion that the basic Asp49 enzymes acquired this toxic activity by changes that directed their catalytic action towards the membrane phospholipids of muscle, whereas the Lys49 proteins, which lost catalytic activity along their evolutionary history due to critical substitutions (Petan, Križaj & Pungerčar, 2007), acquired changes that enabled them to directly alter membrane permeability via their specialized C-terminal region (Lomonte & Gutiérrez, 2011; Fernández et al., 2013).

## Supplemental Information

10.7717/peerj.569/supp-1 Data S1 Raw data of activity tests Phospholipase A_2_ activity, colorimetrically quantified in a microplate reader–Cytotoxic activity, quantified by a UV kinetic assay–Myotoxic activity, quantified by a UV kinetic assay
