# Supplementary material for: Role of enzymatic activity in muscle damage and cytotoxicity induced by Bothrops asper Asp49 phospholipase A2 myotoxins: are there additional effector mechanisms involved?
Source: PeerJ. 2014 Sep 16;2:e569. doi: 10.7717/peerj.569 (PMC4178460; doi:10.7717/peerj.569)
Supplement: Data S1 — Phospholipase A2 activity, colorimetrically quantified in a microplate reader–Cytotoxic activity, quantified by a UV kinetic assay–Myotoxic activity, quantified by a UV kinetic assay [file peerj-02-569-s001.doc]

**Raw data of experiments of manuscript #2014:07:2345:2:0**

**Figure 4**:

panel (D) was generated with the following data:

| **micrograms** | **control toxin** | **SD of control** | **pBPB-modified** | **SD of modified** |
| --- | --- | --- | --- | --- |
| 0.0000 | 0.0000 | 0.0000 | 0.0000 | 0.0000 |
| 1.2500 | 0.1431 | 9.0279e-3 | 4.6667e-3 | 2.3587e-3 |
| 2.5000 | 0.4380 | 0.0551 | 0.0120 | 5.5896e-3 |
| 5.0000 | 0.7701 | 0.0261 | 0.0264 | 7.8437e-3 |

**Figure 5**:

panel (A) was generated with the following data:

| Sample (n = 5) | Creatine knase (U/L) | SD of creatine kinase |
| --- | --- | --- |
| PBS | 136.8 | 42.6 |
| Asp49 control toxin | 8051 | 736 |
| pBPB-modified toxin | 609 | 255.7 |

panel B was generated with the following data:

| Sample (n = 3) | Cytotoxicity (%) | SD of cytotoxicity |
| --- | --- | --- |
| Medium | 0 | 0.5 |
| Asp49 control toxin | 100 | 10 |
| pBPB-modified toxin | 4 | 2 |
